# Ultra-processed Plant Foods: Are They Worse than their Unprocessed Animal-Based Counterparts?

**DOI:** 10.1007/s13668-025-00704-6

**Published:** 2025-10-23

**Authors:** Mariana Del Carmen Fernández-Fígares Jiménez, Miguel López-Moreno

**Affiliations:** 1 School of Medicine, University of Granada, Granada, Spain; 2 Diet, Planetary Health and Performance, Faculty of Health Sciences, Universidad Francisco de Vitoria, Pozuelo, Spain

**Keywords:** Ultra-processed foods, Plant-based alternatives, Plant-based meat analogs, Vegan diet, Plant-based diet, Sustainable diets

## Abstract

**Purpose of the Review:**

This review aims to compare the impact of unprocessed animal foods with ultra-processed plant-based alternatives, particularly plant-based milks, plant-based meat analogs, and margarine, on cardiometabolic risk factors, chronic diseases, and mortality.

**Recent Findings:**

The ultra-processed food category is highly heterogeneous, encompassing products with varying ingredients and nutrient profiles. Plant-based milks, plant-based meat analogs, and margarine, typically classified as ultra-processed foods, differ markedly from their unprocessed animal-based counterparts: they do not contain cholesterol or heme iron, have lower concentrations of saturated fat, sulfur, and branched-chain amino acids, and provide dietary fiber, which is absent in animal-based foods. Replacing dairy milk with soymilk have been shown to reduce total cholesterol (TC), LDL cholesterol (LDL-C), and C-reactive protein (CRP), and is associated with a lower risk of breast cancer. Compared to unprocessed animal-based products, plant-based meat analogs are associated with reductions in TC, LDL-C, body weight, plasma ammonia, and trimethylamine oxide (TMAO). Substituting butter with soft margarine reduces TC and LDL-C, and is associated with a lower risk of cardiovascular events and mortality.

**Summary:**

While ultra-processed plant-based foods are less healthy than whole plant foods, they may offer better cardiometabolic outcomes than unprocessed animal-based products. As transitional tools, products such as plant-based milks, meat analogs, and margarine may facilitate dietary shifts. Public health guidance should reflect these nuances to support realistic, health-promoting transitions.

## Introduction

Ultra-processed foods represent a substantial proportion of modern diets worldwide, raising increasing concern due to their potential association with adverse health outcomes such as obesity, cardiovascular disease (CVD), and all-cause mortality [1]. To better characterize and study these foods, the NOVA food classification system, developed by Monteiro et al., categorizes foods into four groups (unprocessed/minimally processed foods, culinary ingredients, processed foods, and ultra-processed foods) based on the extent and purpose of industrial processing. Within this system, ultra-processed foods are defined as *industrial formulations made by deconstructing whole foods into chemical constituents*,* altering and then recombining them with additives into products that are alternatives to the other three Nova groups and freshly prepared dishes and meals based on them.* The ultra-processed food group encompasses a diverse range of foods, including sugar-sweetened beverages, processed meats, plant-based meat analogs, plant-based milks, and desserts (such as pastries and ice cream) [2].

The NOVA classification system provides a useful framework; however, the category of ultra-processed foods is highly heterogeneous. It includes products with different ingredients and nutrient profiles that may have distinct health effects. For example, processed meatsーwhich are high in sodium, nitrates, saturated fats, and cholesterolーare classified as ultra-processed and have been considered carcinogenic to humans [3]. In contrast, mycoprotein-based foods, which are also categorized as ultra-processed, are high in fiber, low in saturated fat, and cholesterol-free [4]. These favorable dietary characteristics underscore the need to differentiate between nutritionally diverse products within the ultra-processed category and raise the question of how such plant-based foods compare to their unprocessed animal-based counterparts.

Over the past decade, the demand for and availability of plant-based meat analogs and milk alternatives have increased markedly, driven by environmental, ethical, and health concerns [5]. However, the processed nature of plant-based alternatives, as well as their frequent portrayal as nutritionally inferior to animal-based products, remain common barriers to their widespread adoption [5]. While the consumption of unprocessed or minimally processed plant foods is consistently associated with improved health outcomes compared to both animal foods and ultra-processed plant foods [6–8], the health impact of ultra-processed plant foods relative to unprocessed animal foods remains unclear. This narrative review aims to synthesize the current evidence comparing ultra-processed plant-based foods一namely plant-based meat analogs, plant-based milks, and margarine一with their unprocessed animal-based counterparts, with a particular focus on cardiometabolic risk factors, chronic diseases, and mortality.

### Plant-Based Milks

Plant-based milks are non-dairy beverages produced by macerating plant materials in water, typically at proportions ranging from 2% to 15%, followed by homogenization of the mixture [9]. Their nutritional composition varies widely, primarily depending on the main ingredient, which may include grains (e.g., rice, oats), pseudo-cereals (e.g., quinoa), pulses (e.g., soybeans), nuts (e.g., almonds, hazelnuts, walnuts), seeds (e.g., sesame, coconut), or even tubers (e.g., tigernut) [9]. In addition to the type and concentration of the primary plant source, the nutritional profile is also influenced by added ingredients [9]. These may include fortifying substances such as calcium, vitamin D, or vitamin B12, as well as acidity regulators, stabilizers (e.g., gellan gum, guar gum), emulsifiers (e.g., sunflower lecithin), added sugars, vegetable oils, isolated fibers, plant protein isolates, salt, and/or flavorings [9]. The specific ingredients used in each plant-based milk alternative determine its classification within the NOVA system. Particularly, plant-based milks exclusively made with water and the primary plant ingredient are considered “minimally processed”; those that incorporate processed culinary ingredients are classified as “processed” and, if flavorings, cosmetic additives, and/or plant protein isolates are added, then they fall under the “ultra-processed” category. According to an analysis from the UK, 84% of all plant-based milks are classified as ultra-processed (NOVA 4), 14% as processed (NOVA 3), and only 2% as minimally processed (NOVA 1) [10].

**Fig. 1:**
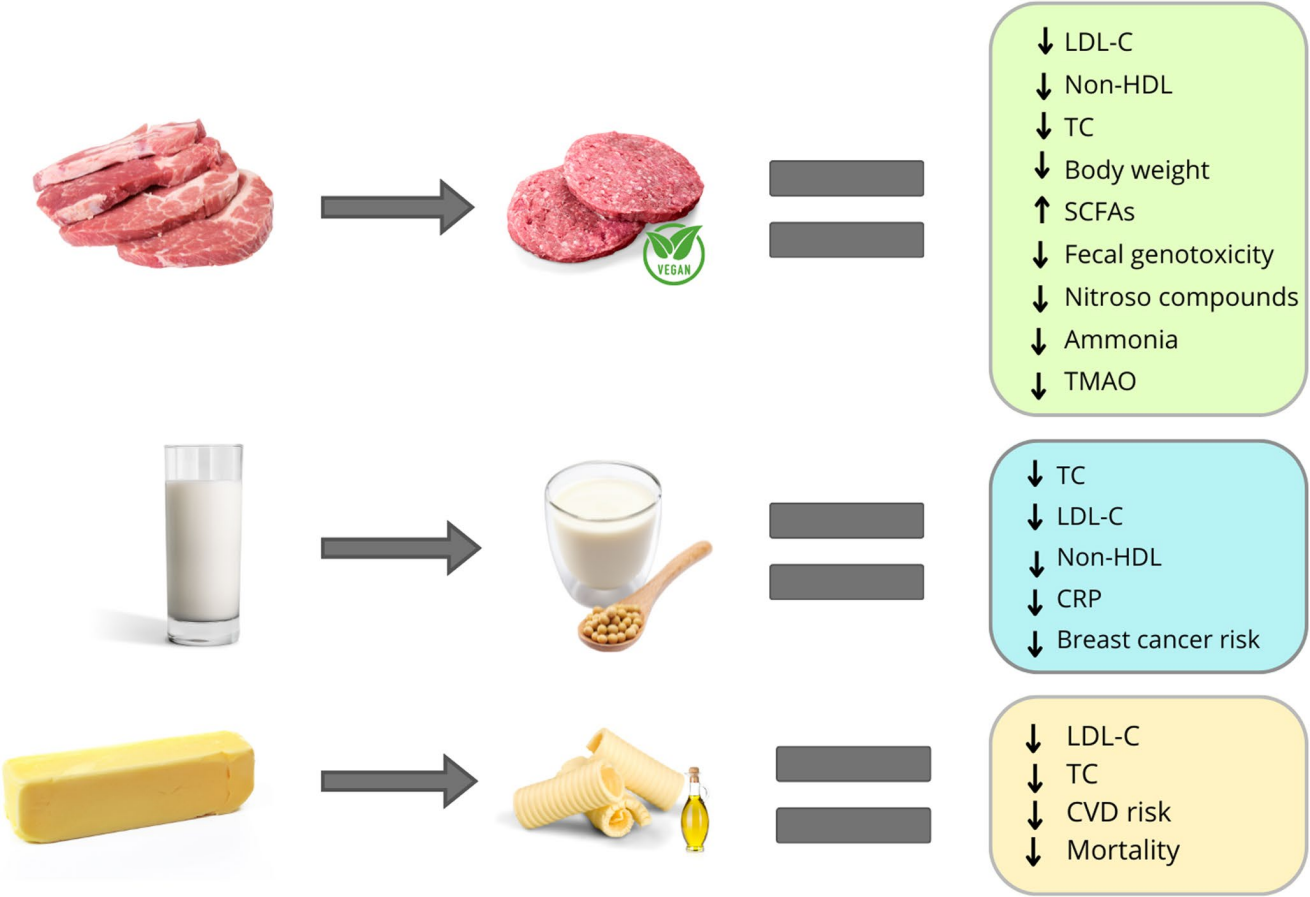
Health effects of substituting unprocessed animal foods with ultra-processed plant-based alternatives. Replacing dairy milk with soymilk leads to reduced levels of TC, LDL-C, and CRP, and it is associated with a lower risk of breast cancer. Substituting meat with plant-based analogs lowers TC, LDL-C, body weight, fecal genotoxicity, nitroso compounds, circulating TMAO, and ammonia, and augments the excretion of SCFAs. Replacing butter with margarine leads to a reduction in TC, LDL-C, CVD risk, and mortality. *Abbreviations*: Low-density lipoprotein cholesterol, LDL-C; non-high-density-lipoprotein cholesterol, non-HDL; total cholesterol, TC; short-chain fatty acids, SCFAs; trimethylamine oxide, TMAO; C-reactive protein, CRP; cardiovascular disease, CVD

Several randomized controlled trials (RCTs) have evaluated and compared the effects of plant-based milk alternatives, particularly soy-based beverages, with cow’s milk. Erlich et al. conducted a systematic review and meta-analysis including 17 RCTs comparing the effects of cow’s milk, considered unprocessed, with soymilk on cardiometabolic risk factors [11]. Of the 17 studies, 9 used ultra-processed soymilk, while the remaining 8 studies did not provide ingredient details [11]. The authors concluded that substituting cow’s milk with soymilk led to a reduction in non-high-density-lipoprotein cholesterol (non-HDL-C), systolic and diastolic blood pressure, as well as small but clinically relevant reductions in low-density-lipoprotein cholesterol (LDL-C) and C-reactive protein (CRP) [11] (Fig. 1). Notably, no significant effect modification was observed based on the presence of added sugars, possibly because soymilk, even when sweetened, has a median sugar content similar to that of cow´s milk (8 g/250 ml vs. 12 g/250 ml, respectively) [11]. Regarding other types of plant-based milks, the number of studies is more limited. In one study, consumption of 0.75–1 L/day of oat milk for 4 weeks reduced TC and LDL-C levels by 4% and 9%, respectively, relative to baseline. However, these changes were not statistically significant when compared to cow’s milk [12].

Concerning chronic disease risk and mortality, soymilk is the most extensively studied plant-based milk in epidemiological studies. A meta-analysis of case-control and cohort studies in the Korean population found that high soymilk consumption was associated with a 33% and 39% reduction in gastric and breast cancer risk, respectively [13]. Similarly, a meta-analysis of cohort studies found that each 242 g serving of soymilk was associated with an 11% lower risk of type 2 diabetes [14]. This meta-analysis included USA studies, showing that the benefits of soy are not limited to the Asian population [14]. Consistent with these findings, analyses of UK Biobank data showed that soymilk consumers had a 30%, 18%, 24% and 43% lower risk of Alzheimer’s disease, cardiovascular events, stroke, and cardiovascular mortality, respectively [15, 16]. Furthermore, in the Adventist Health Study 2 (AHS-2), women who consumed soymilk once a day or more had 56% lower odds of osteoporosis compared with women who did not consume soymilk [17]. Finally, the European Prospective Investigation into Cancer and Nutrition (EPIC) study investigated the impact of ultra-processed foods, including the plant-based alternatives (plant-based milks and meat analogs) subgroup, on the multimorbidity of cancer and cardiometabolic diseases [18]. Ultra-processed foods associated with a higher risk of multimorbidity included animal-based products and both artificially sweetened and sugar-sweetened beverages [18]. In contrast, no association was found with plant-based alternatives [18]. However, the cited studies did not perform substitution analyses directly comparing the health effects of soymilk consumption with those of cow’s milk. This gap was addressed in an analysis within the AHS-2 cohort, which found that replacing one serving of cow’s milk with soymilk was associated with a 32% lower risk of breast cancer [19] (Fig. 1). Notably, in the aforementioned cohort studies, no information was provided regarding the specific ingredients or processing level of the soymilk consumed by participants. However, it is highly likely that most individuals consumed ultra-processed soymilk, given its predominant availability in the market [10].

### Plant-Based Meat Analogs

Plant-based meat analogs refer to food products designed to replicate the texture, flavour, and appearance of animal meat [20]. These are primarily composed of plant protein isolates (such as soy, fava bean, pea, wheat, lupin), which constitute their main ingredients [20]. They typically also include vegetable oils (such as sunflower, rapeseed, coconut oil, or olive oil), salt, and a variety of additives, such as flavorings, thickeners, stabilizers, and emulsifier agents [20, 21]. These ingredients are mixed and processed through extrusion, which may be carried out under a low moisture level (< 30%) to produce dry texturized vegetable proteins, or under a high moisture level (>50%) to yield ready-to-eat meat analogs that do not require rehydration [20]. Examples of plant-based meat analogs include “Beyond Meat” burgers, plant-based mince, “chicken” alternatives, and soy deli slices [21]. From a nutritional standpoint, these products contain no cholesterol, generally have lower saturated fat, and provide higher fiber content compared to meat [21]. They also have lower concentrations of essential amino acids [22]. However, regarding other nutrients, it is difficult to generalize due to the wide variability in ingredients and formulations used across different products [20].

It is important to note that tofu, tempeh, and textured soy are not classified as plant-based analogs. Instead, they are considered “plant-based meat replacements” since their functional and sensory attributes do not resemble those of meat [23]. Plant-based meat replacements are generally categorized as either “minimally processed” or “processed” foods according to the NOVA classification system [2]. Notably, an analysis of the Spanish market found that 93,9% of plant-based meat alternatives, which included plant-based meat analogs and replacements, were ultra-processed (NOVA 4) [21].

Several RCTs have evaluated the effects of plant-based meat analogs on various health outcomes. Particularly, a meta-analysis comprising a total of 7 RCTs found that replacing meat with plant-based analogs (pea, soy, or mycoprotein-based) resulted in reductions of 6% in TC, 12% in LDL-C, and 1% in body weight, [24] (Fig. 1). These effects were primarily driven by studies using mycoprotein-based foods as plant-based analogs [24]. Mycoprotein is a novel protein source produced through biomass fermentation of fungal mycelia, and it contains negligible amounts of saturated fat and higher fiber content compared to other plant-based analogs. Substituting meat and fish with mycoprotein-based analogs has been shown to reduce TC and LDL-C [25, 26], whereas no significant between-group differences have been observed for glucose metabolism parameters, including peripheral insulin sensitivity, glycated hemoglobin (HbA1c), glycemic variability, hepatic insulin sensitivity, postprandial blood glucose, and plasma C-peptide [27].

Soy protein analogs constitute another category of plant-based analogs with promising effects. For instance, one RCT found that replacing 30 g of protein from animal foods with 30 g of protein from soy-based products (including soy nuggets, soy burgers, soy drinks, and soy desserts) led to significant reductions in body weight, TC, non-HDL, LDL-C, and apoB in subjects with metabolic syndrome [28]. In this line, van Nielen et al. showed that an isoproteic and isoenergetic replacement of animal foods (mostly chicken and pork) with soy meat analogues and snacks significantly reduced total and abdominal body fat percentage, as well as plasma triglycerides, TC, and LDL-C in postmenopausal women with metabolic syndrome [29]. Similarly, the Portfolio Diet, which includes soy protein foods (soymilk, soy burgers, soy dogs, soy deli slices), along with almonds, viscous fibers, and plant sterol-enriched foods, has demonstrated a favorable effect on lipid parameters [30]. In particular, compared to a control diet low in saturated fat, which included egg whites and low-fat dairy instead of soy products, the Portfolio Diet led to a significant reduction in LDL-C levels [30]. Notably, the magnitude of LDL-C reduction was comparable to that achieved with statin therapy, suggesting that this dietary approach may be as effective as pharmacological treatment in improving lipid profiles [30].

These lipid-lowering effects have also been observed with plant-based analogs formulated with other isolated plant proteins, such as gluten and pea protein. For instance, in the SWAP-MEAT (The Study With Appetizing Plantfood-Meat Eating Alternatives Trial), replacing unprocessed, grass-fed red meat with Beyond Meat products resulted in significant reductions in LDL-C, body weight, and circulating levels of trimethylamine-N-oxide (TMAO) [31] (Fig. 1). Likewise, in the ECO-Atkins trial, a low-carbohydrate vegan diet that included soy- and wheat-based meat analogs (such as burgers, veggie bacon, deli slices) was compared with a lacto-ovo-vegetarian diet in hyperlipidemic adults [32]. The vegan diet resulted in greater reductions in plasma LDL-C, triglycerides, total cholesterol to HDL-C ratio, and apolipoprotein B to A1 ratio, compared to the lacto-ovo-vegetarian diet, which included low-fat dairy products and egg whites [32, 33]. Finally, Badal et al. found that the substitution of one meat-based meal with a vegan meal, which included a wheat and soy-based meat analog, decreased ammonia production in individuals with cirrhosis [34] (Fig. 1).

### Margarine

Throughout the 20th and early 21 st centuries, margarine was commonly produced using partially hydrogenated oils (PHOs), which contain trans fatty acids [35]. Higher intakes of trans fatty acids have consistently been associated with an increased risk of CVD and mortality [36, 37]. For this reason, the U.S. Food and Drug Administration (FDA) declared in 2015 that PHOs were no longer “Generally Recognized as Safe”, leading to their ban from the U.S. food supply in 2018 [38]. Similarly, in 2019, the European Commission established a maximum limit of 2 g of industrially produced trans fats per 100 g of fat in food products [39]. Since then, margarines have been reformulated using non-hydrogenated vegetable oils, primarily those rich in polyunsaturated fatty acids (PUFAs) [35]. To achieve a semi-solid textured (soft or tub-like) product, emulsifiers are incorporated as additives, resulting in margarine being classified as ultra-processed food [2]. In contrast, butter, naturally high in saturated fatty acids, is classified as a culinary processed ingredient (NOVA 2) [2].

Several RCTs have shown that replacing butter with margarine improves lipid profiles, primarily due to its lower saturated fatty acids and higher concentration of PUFAs [40–42]. For instance, in a crossover study, trans-free margarine intake for 23 days lowered TC and LDL-C levels by 11.1% and 11.3%, respectively, compared with butter [40] (Fig. 1). Notably, this study found that the decreased saturated fat intake during the margarine period was the only variable correlated with dietary responsiveness [40]. Conversely, substituting butter with margarine has not led to improvements in markers of inflammation or endothelial function [43].

In line with these clinical findings, prospective cohort studies have also reported favorable associations. In the Women’s Health Initiative Study, each tablespoon of butter substituted with an equal amount of tub margarine was associated with 8% and 3% decreased risk of myocardial infarction and total coronary heart disease, respectively [44]. Similarly, other US cohorts have reported that substituting butter with margarine was linked to a lower risk of cardiovascular disease and mortality [45].

### Potential Mechanisms

#### Plant Versus Animal Protein

Plant proteins generally contain lower concentrations of essential amino acids, particularly branched-chain amino acids (BCAAs), methionine, and lysine [46]. Higher dietary intakes of these amino acids have been associated with an increased risk of metabolic dysfunction-associated steatotic liver disease [47] and type 2 diabetes [48, 49]. Consistently, intervention trials that restricted dietary methionine and BCAAs reported metabolic benefits, including reduced insulin resistance, increased Fibroblast Growth Factor 2 (FGF-2) plasma concentrations, and upregulated lipogenic gene expression [50–52].

Increased consumption of protein sources rich in essential amino acids, such as dairy milk, also elevates circulating levels of insulin growth factor 1 (IGF-1) [53], which has been causally linked to increased cancer risk [54–56]. This relationship is further supported by studies in individuals with Laron syndrome, who have reduced IGF-1 levels due to a mutation in the IGF-1 receptor, and display a markedly lower incidence of cancer, even in the presence of obesity [57]. The stimulatory effect of dairy milk on the GH-IGF-1 axis may also explain why its higher consumption is associated with increased height [58] and earlier age at menarche [59], both recognized risk factors for breast cancer [60, 61]. In contrast, in children, the consumption of plant-based milks instead of dairy milk has been associated with slightly lower height, but still within the normal range, which may contribute to cancer prevention later in life [62, 63].

Beyond their lower content of growth-stimulating amino acids, plant proteins also provide a distinct profile of bioactive compounds with potential health benefits. Notably, plant protein is characterized by the presence of bioactive peptides that exert hypocholesterolemic, anti-diabetic, anti-inflammatory, and antioxidant actions [64]. For instance, soy protein, which contains the 7 S globulin fraction, has been shown to upregulate hepatic LDL-C receptors, thereby enhancing the clearance of LDL-C from the circulation [65]. This mechanism may explain why soy protein consumption reduces LDL-C concentrations, even when the content of saturated fat and fiber is controlled between diets [66–68].

#### Saturated Fat and Cholesterol

Animal foods contain cholesterol and generally have a higher content of saturated fat than plant-based alternatives [10, 21]. Apart from elevating plasma TC and LDL-C concentrations [69], dietary cholesterol, during cooking, becomes oxidized [70] and, once ingested, it incorporates into plasma lipoproteins [71]. The incorporation of oxidized cholesterol molecules (oxysterols) into LDL particles increases their susceptibility to oxidation, thereby amplifying their atherogenic potential [71]. Therefore, high serum concentrations of 7 beta-hydroxycholesterol and increased oxidation susceptibility of very low-density lipoproteins (VLDL) + LDL are among the strongest predictors of carotid atherosclerotic progression [72]. Concerning saturated fat, it elevates plasma LDL-C concentrations in a dose-response manner by inhibiting LDL receptor activity [73]. Consumption of an animal-based, high-saturated-fat diet also leads to increased levels of skeletal muscle and hepatic triacylglycerols, which trigger the generation of ceramides and diacylglycerols, thereby inducing insulin resistance [74–76]. Furthermore, intake of saturated fat-rich meals impairs postprandial endothelial function [77] and elevates plasma levels of pro-inflammatory bacterial lipopolysaccharide (endotoxin) [78].

Plant-based alternatives, in addition to being cholesterol-free and generally lower in saturated fat, contain proportionally higher amounts of polyunsaturated fatty acids (PUFAs) ーnamely a-linolenic (ALA) and a-linoleic acids (LA)ーcompared with their unprocessed animal-based counterparts [21, 79]. Plant PUFAs exert hypocholesterolemic effects in humans, which may be mediated in part through modulation of the gut microbiota [80]. Particularly, an intervention trial showed a reduction in cholesterol levels when butter was replaced with high-PUFA margarine, and this was correlated with an increased abundance of *Lachnospiraceae*, *Phascolarctobacterium sp.*, and *Eubacterium hallii*, bacterial taxa known to convert cholesterol to coprostanol, a nonabsorbable sterol [80]. In addition, compared to saturated fats, PUFAs reduce proprotein convertase subtilisin/kexin type 9 (PCSK9) activity, leading to an increased number of LDL receptors and consequently enhanced clearance of LDL-C from the circulation [81].

#### Fiber

Some varieties of plant-based analogs and plant-based milks contain dietary fiber, which is absent in animal foods [4, 10, 21]. Oat milk and mycoprotein are, respectively, the plant-based milk and plant-based meat analog with the highest fiber concentrations, mainly in the form of β-glucans [4, 10]. β-glucans are a type of fermentable soluble fiber that have been shown to reduce LDL-C levels [82] and to induce favorable changes in gut microbiota composition and metabolites. The high β-glucans content of mycoprotein may help explain why its consumption increases the abundance of beneficial gut microbes (*Lactobacilli*,* Roseburia*,* and Akkermansi*a), the excretion of anti-inflammatory short-chain fatty acids (SCFAs), and decreases fecal genotoxicity and nitroso compounds compared to meat, potentially contributing to colorectal carcinogenesis prevention [25].

Plant-based milks and meat analogs also contain added isolated fibers, such as guar gum, hydroxypropylmethylcellulose, and xanthan gum, which are used as thickeners and binding agents [10, 20, 21]. In the NOVA system, these are considered “cosmetic additives,” and any food containing them is classified as ultra-processed [2]. However, these isolated fibers have been shown in RCTs to reduce TC and LDL-C levels and improve glycemic control in healthy individuals as well as in those with type 2 diabetes or hypercholesterolemia [83–87]. Additionally, supplementation with guar gum has been found to reduce abdominal pain and improve stool consistency in individuals with irritable bowel syndrome [88, 89].

#### Heme Iron

Meat, unlike plant-based meat analogs, contains heme iron, which is consistently associated with increased risk of cancer [90, 91], type 2 diabetes [92], and CVD mortality [93]. While non-heme iron from plant sources is absorbed in a regulated manner via hepcidin, heme iron is taken up through passive diffusion, which can lead to excessive iron accumulation. This excess iron promotes the formation of hydroxyl radicals, contributing to oxidative stress and increasing the risk of chronic non-communicable diseases [94].

As a substitute for heme iron of non-animal origin, some plant-based meat analogs incorporate leghemoglobin derived from genetically engineered yeast [95]. This phytoglobin, naturally found in the roots of legumes, binds oxygen similarly to human hemoglobin and is used to mimic the taste, smell, and appearance of meat. In these products, leghemoglobin is produced via a safe recombinant method using *Pichia pastoris* yeast genetically modified with the soy leghemoglobin gene and cultivated through a process [95]. According to an in vitro human epithelial cell model, iron uptake from leghemoglobin is comparable to bovine hemoglobin [96]; however, its potential to contribute to iron status has not yet been evaluated in human studies.

#### Other Mechanisms

Other mechanisms may also contribute to the observed differences between plant-based analogs and unprocessed animal-based foods. Plant-based meat analogs contain lower concentrations of advanced glycation end products (AGEs) than their unprocessed, animal-based counterparts [97]. Diets low in AGEs have been associated with reductions in insulin resistance, fasting insulin, TC, and LDL-C, potentially mediated by lower levels of inflammation and oxidative stress [98]. Furthermore, meat may contain carcinogenic polyomaviruses that resist heat inactivation [99, 100], as well as bovine meat factors, a class of infectious circular DNA molecules that have been detected in peritumoral colon and renal cancer tissues [101, 102].

Meat is also a source of carnitine, which is transformed by gut bacterial enzymes into trimethylamine (TMA) through a 2-step process [103]. After absorption into the circulation system, TMA is oxidized to TMAO in the liver [103]. Fish and seafood contain preformed TMAO, which is absorbed more rapidly as it does not require the intervention of the gut microbiota [104]. Elevated plasma TMAO levels have been linked to an increased risk of coronary artery disease, peripheral artery disease, and overall CVD, even after adjustment for traditional risk factors [103]. Reduction in circulating TMAO levels is observed when meat is substituted with plant-based meat analogs, potentially contributing to reduced CVD risk [31].

Unlike plant-based milks, dairy milk contains bioavailable estrogens, leading to increased urinary excretion of estrone, estradiol, and estriol within 1–3 h after its consumption [105]. Pasteurized dairy milk also contains exosomes carrying oncogenic microRNAs (miRs). These include miR-148a-3p, which upregulates estrogen receptor α expression in breast cancer cells and elevates IGF-1 levels, and miR-21-5p, which promotes proliferation and invasion of breast cancer cell lines and is overexpressed in breast cancer tissues compared to normal controls [106].

### Design of Future Studies

Given that ultra-processed plant-based foods appear to exert different health effects compared to ultra-processed animal-based products—and in some cases may even be more favorable than unprocessed animal foods—it is necessary to assess the health impact of ultra-processed foods according to their origin. Within the same food category, such as plant-based milks or meat alternatives, products can range from minimally processed to ultra-processed, with potentially distinct nutritional profiles and health outcomes. However, most epidemiological and clinical studies to date have grouped these products together, without accounting for processing level or specific ingredients. This heterogeneity may obscure important differential effects and limit the ability to draw precise conclusions.

Future research should aim to characterize plant-based foods in greater detail, distinguishing between levels of processing within categories, and ideally incorporating detailed ingredient-level data. Prospective cohort studies and substitution models are particularly needed to assess long-term health outcomes associated with different formulations of plant-based alternatives, including their impact on chronic disease incidence, gut microbiota, and nutrient adequacy. Ultimately, a nuanced approach is essential to avoid overgeneralization and to guide both consumers and policymakers toward healthier and more sustainable dietary choices.

## Conclusions

Public health nutrition guidelines should prioritize plant-based dietary patterns centered on unprocessed or minimally processed whole plant foods, given their consistent association with improved health outcomes compared to both ultra-processed plant-based and animal-based foods. However, for some individuals, adopting such a diet may represent a significant shift, particularly at the initial stages, as it often involves avoiding culturally embedded food choices. In this context, ultra-processed plant-based foods, such as plant-based milks, plant-based analogs, and margarine, may serve as transitional tools, offering more favorable health and environmental profiles than their unprocessed animal-based counterparts. The widespread perception that “natural” or unprocessed foods are inherently healthier can lead to the rejection of plant-based alternatives in favor of animal products such as red meat or dairy. This is concerning, as current evidence suggests that many of these animal foods are more strongly associated with adverse cardiometabolic outcomes and higher chronic disease risk and mortality. Public health messaging should therefore aim to clarify these nuances and encourage dietary transitions that are both health-promoting and realistic.

## Key References

- Fraser GE, Jaceldo-Siegl K, Orlich M, Mashchak A, Sirirat R, Knutsen S. Dairy, soy, and risk of breast cancer: those confounded milks. Int J Epidemiol. 2020;49:1526–37.
  - This cohort study investigated the effect of substituting cow’s milk with soymilk on breast cancer risk.
- Erlich MN, Ghidanac D, Blanco Mejia S, Khan TA, Chiavaroli L, Zurbau A, et al. A systematic review and meta-analysis of randomized trials of substituting soymilk for cow’s milk and intermediate cardiometabolic outcomes: understanding the impact of dairy alternatives in the transition to plant-based diets on cardiometabolic health. BMC Med. 2024;22:336.
  - This meta-analysis included 17 randomized controlled trials that evaluated the replacement of cow’s milk with soymilk on cardiometabolic risk factors.
- Fernández-Rodríguez R, Bizzozero-Peroni B, Díaz-Goñi V, Garrido-Miguel M, Bertotti G, Roldán-Ruiz A, et al. Plant-based meat alternatives and cardiometabolic health: a systematic review and meta-analysis. Am J Clin Nutr. 2025;121:274–83.
  - This meta-analysis included 7 randomized controlled trials that investigated the impact of substituting unprocessed animal foods with plant-based meat alternatives on cardiometabolic risk factors.
- Liu Q, Rossouw J, Roberts M, Liu S, Johnson K, Shikany J, et al. Theoretical Effects of Substituting Butter with Margarine on Risk of Cardiovascular Disease. Epidemiology. 2017;28:145–56.
  - This cohort study evaluated the impact of substituting butter with stick margarine on the risk of clinical myocardial infarction, total coronary heart disease, ischemic stroke, and atherosclerosis-related cardiovascular disease.
- Badal BD, Fagan A, Tate V, Mousel T, Gallagher ML, Puri P, et al. Substitution of One Meat-Based Meal With Vegetarian and Vegan Alternatives Generates Lower Ammonia and Alters Metabolites in Cirrhosis: A Randomized Clinical Trial. Clin Transl Gastroenterol. 2024;15:e1.
  - This intervention study investigated the effect of consuming a vegan meal instead of a meat-containing meal on individuals with cirrhosis.

## Data Availability

No datasets were generated or analysed during the current study.
